# Supplementary material for: Clock transition by continuous dynamical decoupling of a three-level system
Source: Sci Rep. 2018 Oct 4;8:14807. doi: 10.1038/s41598-018-31984-4 (PMC6172288; doi:10.1038/s41598-018-31984-4)
Supplement: Supplementary file 1 — Supplementary Information [file 41598_2018_31984_MOESM1_ESM.pdf]

Supplementary information for:
Clock transition by continuous dynamical decoupling of a three-level system

A.Stark†,[1,2,*] N. Aharon,[3,†] A. Huck,[1] H.A.R. El-Ella,[1] A. Retzker,[3] F. Jelezko,[2,4] and U.L. Andersen[1]

[1] *Center for Macroscopic Quantum States (bigQ), Department of Physics, Technical University of Denmark, Fysikvej, Kongens Lyngby 2800, Denmark*

[2] *Institute for Quantum Optics, Ulm University, Albert-Einstein-Allee 11, Ulm 89081, Germany*

[3] *Racah Institute of Physics, The Hebrew University of Jerusalem, Jerusalem 91904, Israel*

[4] *Center for Integrated Quantum Science and Technology (IQ^{st}), Ulm University, 89081 Germany*

S1. SETUP CONFIGURATION

We used a standard home-build confocal microscope to address individual NV centres in a diamond sample. The diamond sample was a 98.9 % ^{12}C electronic grade substrate with a 40 µm thick and 99.8 % ^{12}C layer grown on top by chemical vapour deposition. The growth layer has a low concentration nitrogen doping forming individual NV centers which can be optically resolved in our confocal microscope. The top side of the diamond was attached to a glass slide with a lithographically deposited microwave structure to enable close vicinity to the NV center and hence efficient microwave driving. The glass structure with the diamond was attached to a printed circuit board (PCB) sample holder with SMA connectors for microwave field delivery. The PCB board was mounted on a xyz micrometer stage to position the sample relative to the microscope piezo scanner holding the Olympus UPlanFL N 100X/1.30 objective. Our xyz piezo scanner is a P-527.3CD from Physik Instrumente with an E-725.3CD Digital Piezo Controller.

The excitation light is provided by a Coherent Verdi G2-SLM OPS 532 nm laser pulsed with an AOM from Brimrose. In our setup, we separate the red fluorescence from the green illumination using a dichroic mirror FF552-Di02-25x36 from Semrock and detect the signal with an avalanche photon diode (APD), SPCM-AQRH from Excelitas Technologies. The electrical signal is either recorded by a custom configured XEM6310 LX45 FPGA from Opal Kelly, with a 950 MHz detection bandwidth (for fast counting), or with a PCI-6232 X-series card from National Instruments (for slow counting). The hardware triggered confocal scanning operation as well as the ODMR measurements are controlled with the NI PCI-6232 card.

The creation of the microwave field is either realised with a Rohde&Schwarz SMR20 microwave signal generator (for ODMR measurements) or with an arbitrary waveform generator AWG70002A from Tektronix (borrowed for a short period). In both cases, the microwave field was amplified by a Mini-Circuits ZHL-16W-43 S+ high power amplifier.

All the instruments are remote controlled by a PC, which handles the sequence control and data acquisition. The powerful software suite Qudi [1] serves as the operational tool to establish the control over all hardware components and performs the measurements.

The NV selected for this experiment was about 1 µm deep in the diamond and about 10 µm away from the microwave structure. We performed this experiment at a magnetic field strengh of 358 G in the vicinity of the excited state level anti-crossing [2–4], in which case the nitrogen nuclear spin becomes polarised within a few optical pumping cycles. The static magnetic field was created by two neodymium magnets in a quadrupole configuartion and aligned along the quantization axis. The magnets were attached (with a 3D printed holder) to a three axes NRT150/M 150 mm motorised linear translation stage with a BDC103 three channel controller from Thorlabs. In addition, a Thorlabs motorised rotation state with a TST001 driver controled the angular movement of the magnet. For an excitation power of about 250 µW, measured before the objective, we obtain a fluorescence signal of around 200 kcounts/s at zero magnetic bias fields.

* Email of corresponding author: astark@fysik.dtu.dk
† These authors contributed equally

S2. SYSTEM PARAMETERS

A. Pulsed ODMR measurements

We performed high resolution pulsed ODMR measurements on both resonances, ν_1 and ν_2, to identify potential coupling to adjacent ^{13}C nuclear spins. Within the full width half maximum of a Lorentzian fit, no additional couplings to ^{13}C nuclear spins are visible (cf. Fig. S1). The average of both transition frequencies yields $(\nu_1+\nu_2)/2 = (2870.7005 \pm 0.008)$ GHz, which corresponds to the zero-field splitting parameter $D = 2.87$ GHz and hence indicats a well aligned bias magnetic field.

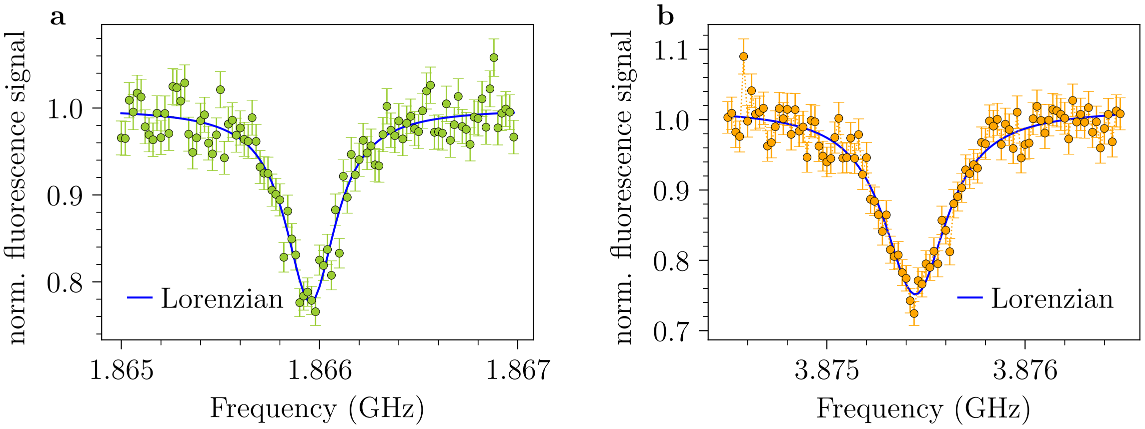

Figure S1. Pulsed ODMR measurement. (a) For the lower transition, a 3.3 µs long π-pulse was applied to invert the population. A Lorenzian fit yields an FWHM of (306 ± 23) kHz at $\nu_1 = (1865.955 \pm 0.006)$ MHz. (b) For the upper transition, a 2.7 µs long π-pulse was applied, with an obtained FWHM of (387 ± 24) kHz at $\nu_2 = (3875.446 \pm 0.006)$ MHz. The error bar in both plots corresponds to the normalised Poissonian error of the photon count signal.

B. Coherence times of the Rabi drives

To ensure that both transitions, ν_1 and ν_2, experience the same Rabi frequency, Ω, we had to adjust the drive strength on the transitions individually. The inset of Fig. S2 shows the dependency of the Rabi frequency, Ω, on the amplitude of the signal generator. It becomes apparent that the lower transition, ν_1 requires a larger drive amplitude to yield a similar Rabi frequency as on ν_2. The reasons for the imbalances can be manifold and are discussed in Sec. S4.

The largest coherence time at ν_1 was achieved for a drive of $\Omega/2\pi = (1.366 \pm 0.006)$ MHz with $T_2^{\Omega(\nu_1)} = (62 \pm 12)$ µ. For the upper transition at ν_2, a drive of $\Omega/2\pi = (2.816 \pm 0.006)$ MHz yields $T_2^{\Omega(\nu_2)} = (159 \pm 24)$ µs.

The coherence time of the individual transitions is rather different. In order to record the coherence times, we first determined the correct Rabi frequency for a given drive amplitude. Then we measured the coherence time by deliberately undersampling the obtained Rabi frequency in order to substantially reduce the total measurement time (for more details see Sec. S3).

The most relevant characteristics can be extracted from the coherence time measurements presented in Fig. S2. Each driving field, Ω_i, posses a noise component $\delta\Omega_i$. The amplitude of the driving noise depends on the strength of the drive and its scaling with amplitude depends on the measurement configuration (the frequency dependent amplification and attenuation properties of the components). The coherence time curves of both transitions coincide for small Rabi frequencies, as the external magnetic noise, δB, constitutes to be the main dephasing contribution in this regime. By increasing the drive a larger dressed state energy gap can be created protecting the system from magnetic noise, δB. At the same time drive fluctuations become more prominent and dominate from a certain drive value on the noise contribution. The maximum coherence time indicates a balanced situation, where the dressed states of the drive experience similar noise contributions from the drive and the environment. Increasing the drive further leads to a non-linear increase of the drive noise and hence the coherence time decreases.

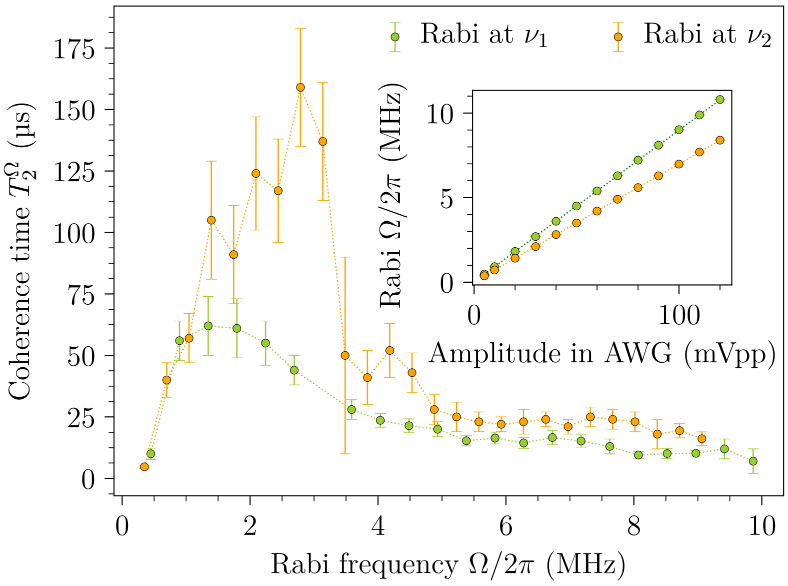

Figure S2. Rabi measurements at two transitions, $\nu_1 = (1864.942 \pm 0.019)$ MHz and at $\nu_2 = (3876.467 \pm 0.016)$ MHz. The main figure shows coherence times of Rabi oscillations, T_2^Ω, as a function of Rabi drive, Ω. The difference in T_2^Ω between both transitions originates mainly from a different drive noise characteristic at ν_1 and ν_2. The error bars are standard deviations, ΔT_2^Ω, from the fit $S(t) = A\cos(\Omega t)\exp\left(-t/T_2^\Omega\right)$ to the decaying Rabi signal. Inset: measurement of the Rabi frequency for different amplitudes of the signal generator. A linear fit to the slope yields $m_{\nu_1} = (89.68 \pm 0.10)$ kHz/mV and $m_{\nu_2} = (69.72 \pm 0.05)$ kHz/mV. The error bars correspond to $\Delta\Omega$.

The drastic difference in the appearance of the coherence time curves indicates a frequency dependent noise of the drive fields, respectively for ν_1 and ν_2. In contrast to this, the magnetic noise appear to have an equal impact on both transitions, which will be further addressed in the following sections.

C. Ramsey measurements

We performed a Ramsey measurement on both transitions to identify that external and internal magnetic noise have the same impact on the $m_s = -1$ and $m_s = +1$ spin states. The measurement results presented in Fig. S3 demonstrate that within the error bars both transitions have an identical pure dephasing time, and the dephasing time averaged over both transitions is $T_2^* = (1.78 \pm 0.24)$ µs.

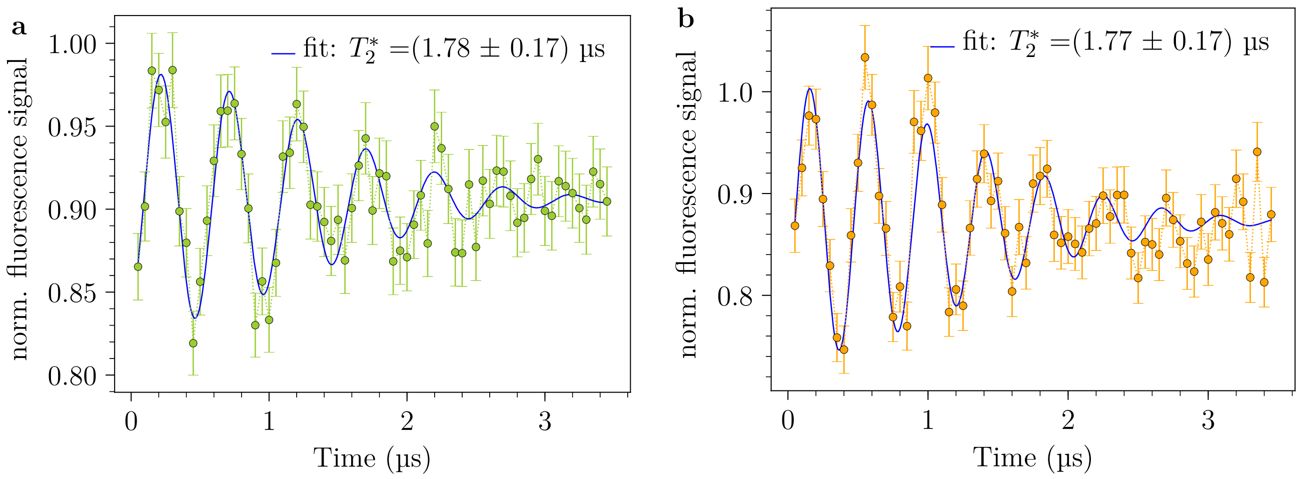

Figure S3. Ramsey coherence time measurement. The $\pi/2$ pulses in the scheme were deliberately detuned by 2 MHz to determine the coherence time, T_2^*, in the decreasing oscillating signal more accurately. The curves were fitted to the function $\propto \sin(\omega t + \phi) \exp\left\{-(t/T_2^*)^\beta\right\}$, where $\beta = 2.0 \pm 0.1$ was obtained for both curves. Hence, Gaussian uncorrelated noise can be assumed. (a) shows the lower transition and (b) the upper one, where both were measured at the ODMR frequency (+2 MHz) given in Fig. S1. The error bar in both plots corresponds to the normalised Poissonian error of the photon count signal.

D. Hahn Echo measurements

We also carried out a coherence time measurement with a Hahn Echo scheme (cf. Fig. S4). Within the error bar, both transitions yield the same coherence time, T_2. An averaged coherence time of $T_2 = (215 \pm 31)$ µs can be stated for the system and hence we can consider that the magnetic noise acting on both the $m_s = -1$ and $m_s = +1$ states is identical.

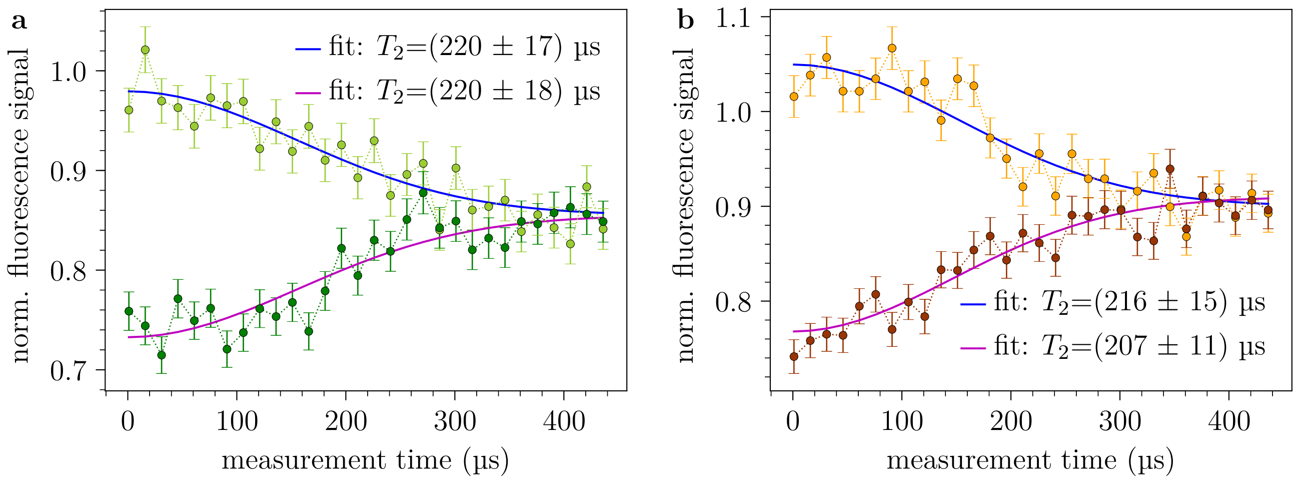

Figure S4. Hahn Echo coherence time measurement. The last pulse in the Hahn Echo measurement was either projecting the measurement to the bright ($\pi/2$-pulse, top curve) or to the dark ($3\pi/2$-pulse, lower curve) spin state of the NV center. Identifying the decoherence level to which both curves decaying to, increases the accuracy of the fit. The coherence time was obtained by fitting to $\propto \exp\{-(t/T_2)^2\}$ for (a) the lower and (b) the upper transition, corresponding to the ODMR measurement in Fig. S1. The error bar in both plots represent the normalised Poissonian error of the photon count signal.

S3. MEASUREMENT PROCEDURE

In this section, we want to explain the technique applied to measure the coherence times, which are shown in the main text. We would like to emphasise that a compromise was needed between the measurement time, the number of data points and the resulting error bars, which are responsible for the larger error bars in Fig. 4 of the main text.

Almost all measurement curves in this experiment can be represented by $S(t) = A\cos(2\pi\nu_{\mathrm{Rabi}})\exp\left(-t/T_2^{\Omega_B}\right)$, where $\nu_{\mathrm{Rabi}} = \Omega_B/2\pi$ is the Rabi frequency and $T_2^{\Omega_B}$ is the coherence time depending on the detuning, Δ, in the double drive (see Fig. 2 in the main text).

Consequently, we could simply measure the whole trace with a resolution of about 4 points per period. This would require a sampling frequency of $\nu_{\mathrm{samp}} = 4 \cdot \nu_{\mathrm{Rabi}} \approx 13\,\mathrm{MHz}$, which corresponds to a time step of $\sim 77\,\mathrm{ns}$.

In Fig. S5 the Rabi period is sampled with roughly 10 points, resulting in a good estimate of the frequency (relative error of about $\sim 0.15\%$). Hence, to sample a full decay with a length up to $250\,\mathrm{\mu s}$ would require to record (at 4 points per period) about 3250 points. Then a signal to noise of about 10 requires more than 1 day of measurement for one detuning value. Since in this case it becomes impractical to carry out the experiment, we significantly reduced the measurement time by acquiring the data in a different way as explained in the following.

The simplest idea is to sample the oscillation at every period ($\nu_{\mathrm{samp}} = \nu_{\mathrm{Rabi}}$) to extract directly the exponential decay from the data. However, this would result in larger fit errors since the exponential fit requires a much smaller noise component on the data to yield a good coherence times estimate. Moreover, if the Rabi frequency, ν_{Rabi} was measured with 0.15% accuracy (this corresponds to an absolute error of $\pm 5\,\mathrm{kHz}$), it would mean that there might be oscillations in the measured time trace with frequencies $\nu_{\mathrm{osci}} \lesssim$ resulting from an incorrect estimation of the Rabi frequency. At these time scales the frequency component of a decaying signal cannot be extracted accurately since only a part of the full period could be sampled. These reasons required to refine the measurement technique.

In this work we choose to deliberately undersample the known Rabi frequency by a specific amount to measure effectively a slowly oscillating decaying image frequency. Such time traces show a better robustness in the fitting algorithm and are eventually a compromise in measurement time and the resulting error bars in the fit.

We have included Fig. S6 to show an example of an undersampled time trace from which we extracted the coherence time. As a result, we could drastically reduce the amount of measurement points and the undersampled time trace

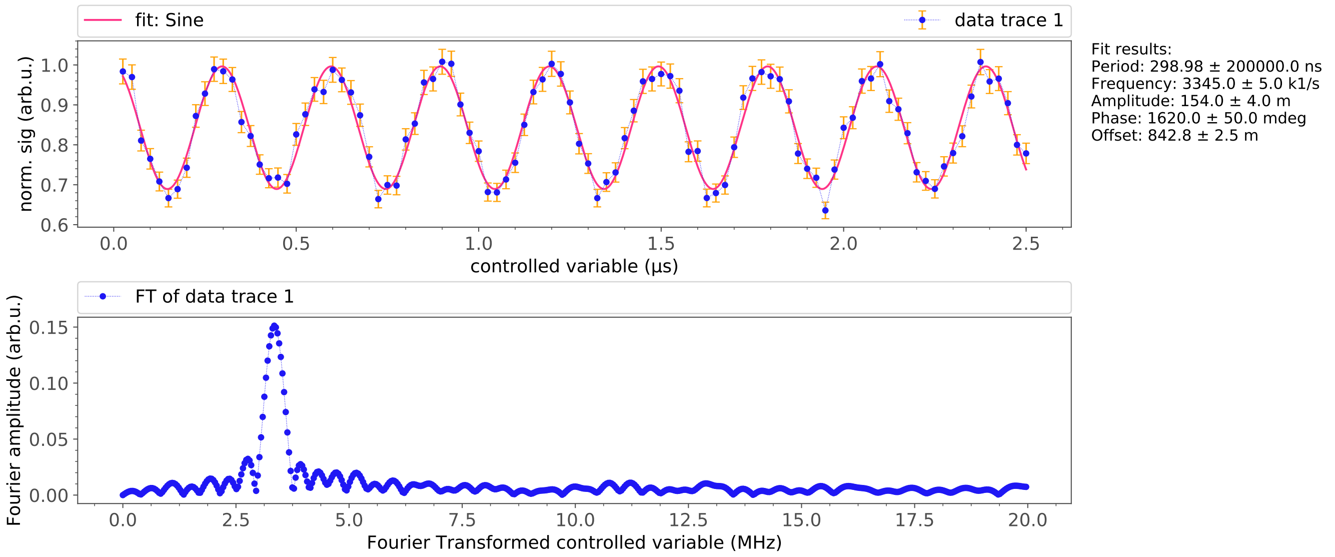

Figure S5. Measurement of a Rabi drive of the bright state. The top graph shows the accumulated normalised fluorescence count signal (normalised to the count of the bright state). In the graph below is the Fourier transform of the top graph. On the side of the graph the fit results of a sinusoidal function are displayed.

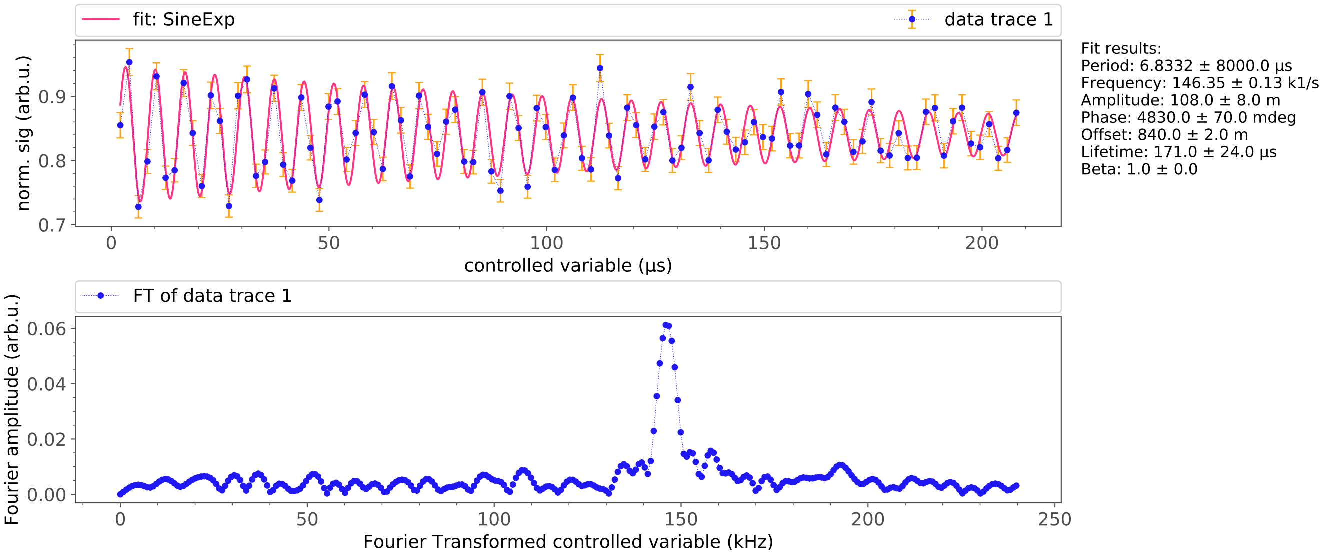

Figure S6. Measurement for an arbitrary chosen detuning point at $\Delta = 28\,\mathrm{MHz}$ to display the undersampled idea of the situation. The top graph shows the normalised fluorescence time trace, whereas the bottom graph displays its Fourier transform. In the side panel, the fit results of a single exponential sinusoidal decay are summarised. Here, the frequency does not correspond to the actual Rabi frequency of the drive but due to the deliberate undersampling an image frequency is obtained.

was averaged for about 1 hour yielding a measurement result displayed in Fig. S6.

S4. ESTIMATED NOISE CHARACTERISTICS

In this section, we try to identify the components contributing to drive noise of the fields. Based on the Ramsey and the Hahn Echo measurements, we can assume identical external magnetic noise at both transition frequencies, ν_1 and ν_2. In continuous dynamical decoupling the NV is permanently subjected to strong drive fields and therefore, drive noise will be the strongest noise contribution our scheme. We did not have access to a fast oscilloscope and because of that could not measure the fast noise components directly. However, based on conservative estimates we would like to state the various noise contributions in our system.

The drivings field in our work are created by the Tektronix AWG70002A. The maximal output amplitude for this device is 0.5 Vpp (or 1 Vpp by including the inverted channel) with a DAC accuracy of 8bit, yielding a maximum precision of $0.5\,\mathrm{Vpp}/2^8 \approx 2\,\mathrm{mVpp}$ or 1 mV for the amplitude of an oscillating signal. Consequently, a sinusoidal field with an amplitude of 0.25 V (0.5 Vpp) has an accuracy of $(250 \pm 1)\,\mathrm{mV}$, which yields a relative error of 0.78 %. The vendor specifies the amplitude accuracy to $\pm 10\,\mathrm{mVpp}$ [5], which increases the relative error to 2 %, indicating that besides the DAC inaccuracy, other noise sources exist. Therefore, the application of 4 sinusoidal fields at different frequencies can have at maximum 1/4 of the total amplitude noise, if the noise is equally distributed on all channels (which is not necessarily the case). Thus, one frequency component is accurate up to $(62.5 \pm 1.3)\,\mathrm{mV}$, yielding a relative amplitude error of 2 %.

A field of 62.5 mV (= 125 mVpp) would create (see the inset of Fig.1 in the main text) a Rabi drive of the order of 10 MHz. Increasing the drive field above that value caused heating problems, as a lot of power is dissipated in the microwave structure causing a drastic decrease in ODMR contrast and fluorescence signal. To prevent that from happening we lowered the amplitude. We found a balance between the thermal effects and the maximum obtainable Rabi frequency for fields of the order of 40 mVpp, if 4 fields are used simultaneously, resulting in a total amplitude of 160 mVpp. Given these limitation on the drive fields, the relative noise for each frequency component is on average 6.25 %.

Moreover, the amplitude noise of the AWG is correlated to its base clock, which introduces correlated (amplitude) noise on a µs timescale and may very well be responsible for frequency dependent (fast) noise imbalances, translated to the dressed states of the considered three-level system (see main text Fig. 4). The origin of this noise character can be grasped by investigating how data becomes sampled in an AWG.

Modern, very fast AWGs (with sample rates exceeding 1GS/s) do not process each sample directly as the rate the data is read exceeds the speed of common semiconductor memories [6]. To circumvent this problem, data is processed in parallel and then multiplexed inside the high-speed digital logic that drives the DAC. However, the multiplexing operation is directly bound to the internal clock of the AWG, which is usually of the order of 10MHz and thus creates correlations on that time base. Any errors in the timing of the internal clock edge translates directly into timing errors in the analogue output, which reflects phase noise and is immediately detectable by amplitude fluctuations. This is a noise source, which adds to the DAC inaccuracy and creates (among other effects) the total noise floor of the device. Eventually, a high-speed oscilloscope would be the preferential choice to identify fast noise on the individual frequency components. The clock stability of the device is denoted by 1 ppm for 10 MHz [5], i.e. the total frequency variations are on the order of $(10\,\mathrm{MHz}/10^6) = \pm 10\,\mathrm{Hz}$.

On top of this, we also assume a frequency dependent noise, since the output of the amplifier is not flat over a range of $\nu_2 - \nu_1 \approx 2\,\mathrm{GHz}$. Moreover, the microwave structure experiences frequency dependent attenuation of the applied drive fields leading to (among other contributions) different Rabi frequencies for ν_1 and ν_2 at the same amplitude output (cf. the inset of Fig.1 in the main text).

All these various noise contributions have a strong impact on the drive of the NV system as they cause the dressed states to fluctuate. Therefore it becomes a crucial task in CCD to reduce the impact of noise, introduced by multi-frequency drive fields, by creating robust states, which suffer less from drive noise contributions. By demonstrating robustness to all these noise sources underlines the importance of the present work.

S5. ADDITIONAL EXPERIMENTAL ERRORS

In this section we analyse the effect of frequency and phase errors of the driving fields. These errors have a negligible influence on our experimental setup since the largest contribution results from amplitude noise (as described in Sec. S4). However, we want to give in the following a qualitative analysis of these errors in the case of an on-resonance driven Λ system. Specifically, we consider the ideal (noiseless) three-level Hamiltonian

$$H = \omega_0 S_z^2 + g\mu_B B S_z + \sqrt{2}\Omega\Big(\cos^{(\omega_0+g\mu_B)t}(|0\rangle\langle+1| + |+1\rangle\langle 0|)\Big) + \sqrt{2}\Omega\Big(\cos^{(\omega_0-g\mu_B)t}(|0\rangle\langle-1| + |-1\rangle\langle 0|)\Big), \quad (1)$$

which when moving to the interaction picture (with respect to $H_0 = \omega_0 S_z^2 + g\mu_B B S_z$) and making the RWA ($\omega_0 \pm g\mu_B \gg \Omega$) results in $H_I = \Omega S_x$. The dressed states, the eigenstates of H_I, are robust to magnetic noise, $\delta B S_z$. When $\Omega \gg \delta B$, the leading effect of the magnetic noise is $\frac{(\delta B)^2}{2\Omega}$.

A. Frequency noise

Denote by δ_1 and δ_2 the noise of the frequencies of the first and second drives respectively. The driving Hamiltonian is given by

$$H = \omega_0 S_z^2 + g\mu_B B S_z + \sqrt{2}\Omega\Big(\cos^{(\omega_0+g\mu_B+\delta_1)t}(|0\rangle\langle+1| + |+1\rangle\langle 0|)\Big) + \sqrt{2}\Omega\Big(\cos^{(\omega_0-g\mu_B+\delta_2)t}(|0\rangle\langle-1| + |-1\rangle\langle 0|)\Big). \quad (2)$$

The Hamiltonian in the interaction picture (with respect to $H_0 = \omega_0 S_z^2 + g\mu_B B S_z + \delta_1 |+1\rangle\langle+1| + \delta_2 |-1\rangle\langle-1|$) is given by $H_I = \Omega S_x - \delta_1 |+1\rangle\langle+1| - \delta_2 |-1\rangle\langle-1|$. Up to first order in δ_1 and δ_2, the eigenvalues are equal to $\{+\Omega - \frac{\delta_1+\delta_2}{4}, -\Omega - \frac{\delta_1+\delta_2}{4}, -\frac{\delta_1+\delta_2}{2}\}$. The dressed states are therefore sensitive to frequency noise at first order.

B. Correlated phase noise

Here we assume that both driving fields have the same phase noise denoted by δ. The driving Hamiltonian is given by

$$H = \omega_0 S_z^2 + g\mu_B B S_z + \sqrt{2}\Omega\Big(\cos^{(\omega_0+g\mu_B)t+\delta}(|0\rangle\langle+1| + |+1\rangle\langle 0|)\Big) + \sqrt{2}\Omega\Big(\cos^{(\omega_0-g\mu_B)t+\delta}(|0\rangle\langle-1| + |-1\rangle\langle 0|)\Big). \quad (3)$$

The Hamiltonian in the interaction picture (with respect to $H_0 = \omega_0 S_z^2 + g\mu_B B S_z$) and in the basis of $\{|0\rangle, |B\rangle = \frac{1}{\sqrt{2}}(|+1\rangle + |-1\rangle), |D\rangle \frac{1}{\sqrt{2}}(|+1\rangle - |-1\rangle)\}$ is given by $H_I = \frac{\Omega}{\sqrt{2}}(e^{-i\delta}|0\rangle\langle B| + h.c.)$. Because δ is generally time-dependent, the basis of the dressed states, which is determined by δ, is time dependent. In this case, the coupling rate between the instantaneous dressed states is $\sim \frac{d\delta}{dt}$. It the limit of a slow noise, $\frac{d\delta}{dt} \ll \Omega$, the coupling results in a dephasing rate of $\sim (\frac{d\delta}{dt})^2/\Omega$. In the limit of a fast noise, $\frac{d\delta}{dt} \sim \Omega$, the drive fails to create robust dressed states.

C. Non-correlated phase noise

We now assume that the driving Hamiltonian is given by

$$H = \omega_0 S_z^2 + g\mu_B B S_z + \sqrt{2}\Omega\Big(\cos^{(\omega_0+g\mu_B)t+\delta_1}(|0\rangle\langle+1| + |+1\rangle\langle 0|)\Big) + \sqrt{2}\Omega\Big(\cos^{(\omega_0-g\mu_B)t+\delta_2}(|0\rangle\langle-1| + |-1\rangle\langle 0|)\Big). \quad (4)$$

Proceeding as above and assuming that $\delta_2 = \delta_1 + \Delta$, the Hamiltonian, up to first order in Δ, in the interaction picture (with respect to $H_0 = \omega_0 S_z^2 + g\mu_B B S_z$) and in the basis of $\{|0\rangle, |B\rangle, |D\rangle$ is given by $H_I = \frac{\Omega(2+i\Delta)}{2}(e^{-i\delta_1}|0\rangle\langle B| + h.c.) + \frac{\Omega\Delta}{2}(-ie^{-i\delta_1}|0\rangle\langle D| + h.c.)$. Δ introduces both (first order) noise in the coupling between $|0\rangle$ and $|B\rangle$ (a dephasing rate of $\sim \Omega\Delta$, and a coupling between $|0\rangle$ and $|D\rangle$, which effects the robustness to magnetic noise by introducing an amplitude mixing of $\sim \Omega\Delta$ between $|B\rangle$ and $|D\rangle$.

S6. SIMULATIONS

For the simulations we assumed a pure dephasing time of $T_2^* = 2\,\mu s$, and under a Hahn Echo pulse, a coherence time of $T_2 = 230\,\mu s$. In our model, the magnetic noise has two components. One component, B_r, is a random field that is static within each experiment, but has a different value in different experiments, and a second component, $B(t)$, which is an Ornstein-Uhlenbeck process [7, 8] with a zero expectation value, $\langle B(t)\rangle = 0$, and a correlation function $\langle B(t)B(t')\rangle = \frac{c\tau}{2}\exp(-\gamma|t-t'|)$. B_r is normally distributed with a width of $\sigma \approx \frac{0.99\cdot\sqrt{2}}{T_2^*}$. An exact simulation algorithm [9] was employed to realise the Ornstein-Uhlenbeck process, which according to

$$B(t+\Delta t) = B(t)e^{-\frac{\Delta t}{\tau}} + n\sqrt{\frac{c\tau}{2}\left(1 - e^{-\frac{2\Delta t}{\tau}}\right)}, \quad (5)$$

where n is a unit Gaussian random number. The correlation time of the noise was set to $\tau = 1/\gamma = 15\,\mu s$, where the diffusion constant is given by $c \approx \frac{2}{T\tau^2}$. Here we choose the parameter $T = 186\,\mu s$ to obtain in the simulations a coherence time of $T_2 = 230\,\mu s$. For pure dephasing, we simulated the Hamiltonian

$$H = \frac{\omega_0}{2}\sigma_z + \Big(B_r + B(t)\Big)\sigma_z, \quad (6)$$

where we used $\omega_0/2\pi = 15.92\,MHz$ and the qubit was initialised to $|\uparrow_x\rangle$. For the Hahn echo pulse we run the same simulation but flipped the sign of $(B_r + B(t))\sigma_z$ at $t = 115\,\mu s$. These simulations confirmed that our noise model results in a pure dephasing time of $T_2^* = 2\,\mu s$ and a Hahn echo time of $T_2 = 230\,\mu s$.

Driving fluctuations were also modeled by an Ornstein-Uhlenbeck process with a zero expectation value. Indicated by the experimental results, we chose a long correlation time of $\tau_\Omega = 5000\,\mu s$. In the simulations of the experiment, in order to reproduce the location of the peak of the coherence time (the optimal detuning) and the coherence

time under a large detuning (single Λ drive limit), we set the relative amplitude errors of the drives according to $\delta_{\Omega_D} = 0.33\%$ and $\delta_{\Omega_B} = 3.94\delta_{\Omega_D}$. In the simulations of the improved scheme we assumed $\delta_{\Omega_D} = \delta_{\Omega_B} = 0.5\%$. The diffusion constant is given by $c_{\Omega_i} = 2\delta_{\Omega_i}/\tau_\Omega$.

All simulations were preformed in the lab frame and no approximations of the driving Hamiltonian were applied, resulting in

$$\begin{aligned} H_d =& 2\Omega(1+\delta_{\Omega_D}(t))\Big(\cos((\omega_1-\Delta_0)t)-\cos((\omega_2-\Delta_0)t)\Big)S_x \\ &+2\Omega(1+\delta_{\Omega_B}(t))\Big(\cos((\omega_1+\Delta-\Delta_0)t)+\cos((\omega_2+\Delta-\Delta_0)t)\Big)S_x, \end{aligned} \tag{7}$$

and magnetic noise Hamiltonian of

$$H = \Big(B_r + B(t)\Big)S_z. \tag{8}$$

[1] J. M. Binder, A. Stark, N. Tomek, J. Scheuer, F. Frank, K. D. Jahnke, C. Müller, S. Schmitt, M. H. Metsch, T. Unden, T. Gehring, A. Huck, U. L. Andersen, L. J. Rogers, and F. Jelezko, SoftwareX **6**, 85 (2017).
[2] V. Jacques, P. Neumann, J. Beck, M. Markham, D. Twitchen, J. Meijer, F. Kaiser, G. Balasubramanian, F. Jelezko, and J. Wrachtrup, Physical Review Letters **102**, 057403 (2009).
[3] V. Ivády, K. Szász, A. L. Falk, P. V. Klimov, D. J. Christle, E. Janzén, I. A. Abrikosov, D. D. Awschalom, and A. Gali, Physical Review B **92**, 115206 (2015).
[4] D. A. Broadway, J. D. Wood, L. T. Hall, A. Stacey, M. Markham, D. A. Simpson, J.-P. Tetienne, and L. C. Hollenberg, Physical Review Applied **6**, 064001 (2016).
[5] Tektronix, "AWG70000a Arbitrary Waveform Generator Datasheet | Tektronix," (2016).
[6] Keysight Technologies, "The ABC's of Arbitrary Waveform Generation - An Application Note," (2006).
[7] M. C. Wang and G. E. Uhlenbeck, Reviews of Modern Physics **17**, 323 (1945).
[8] R. Hanson, V. V. Dobrovitski, A. E. Feiguin, O. Gywat, and D. D. Awschalom, Science **320**, 352 (2008).
[9] D. T. Gillespie, Physical Review E **54**, 2084 (1996).
